# The impact of a minimal smoking cessation intervention for pregnant women and their partners on perinatal smoking behaviour in primary health care: A real-life controlled study

**DOI:** 10.1186/1471-2458-8-325

**Published:** 2008-09-22

**Authors:** Torbjørn Øien, Ola Storrø, Jon A Jenssen, Roar Johnsen

**Affiliations:** 1 Department of Public Health and General Practice, Faculty of Medicine, Norwegian University of Science and Technology, N-7489 Trondheim, Norway

## Abstract

**Background:**

There is a demand for strategies to promote smoking cessation in high-risk populations like smoking pregnant women and their partners. The objectives of this study were to investigate parental smoking behaviour during pregnancy after introduction of a prenatal, structured, multi-disciplinary smoking cessation programme in primary care, and to compare smoking behaviour among pregnant women in the city of Trondheim with Bergen and Norway.

**Methods:**

Sequential birth cohorts were established to evaluate the intervention programme from September 2000 to December 2004 in primary care as a part of the Prevention of Allergy among Children in Trondheim study (PACT). The primary outcome variables were self reported smoking behaviour at inclusion and six weeks postnatal. Data from the Medical Birth Registry of Norway (MBR) were used to describe smoking cessation during pregnancy in Trondheim, Bergen and Norway 1999–2004.

**Results:**

Maternal smoking prevalence at inclusion during pregnancy were 5% (CI 95% 4–6) in the intervention cohort compared to 7% (CI 95% 6–9), p = 0.03, in the control cohort. Of the pre-pregnancy maternal smokers 25% (CI 95% 20–31) and 32% (CI 95% 26–38), p = 0.17, were still smoking at inclusion in the intervention and control cohorts, respectively. Six weeks postnatal 72% (CI 95% 59–83) and 68% (CI 95% 57–77), p = 0.34 of the maternal smokers at inclusion still smoked. No significant difference in paternal smoking between the cohorts was found after the intervention period. Data from the MBR showed a significantly higher proportion of women who stopped smoking during pregnancy in Trondheim than in Bergen in 2003 and 2004, p = 0.03 and < 0.001, respectively.

**Conclusion:**

No impact on parental smoking behaviour between the cohorts was observed after the smoking intervention programme. Of the women who stopped smoking during pregnancy most of them stopped smoking before the intervention. However, we observed a significantly higher quitting rate in Trondheim than in Bergen in 2003 and 2004 which may have been facilitated by the supplemental attention on smoking behaviour the PACT study initiated.

## Background

Smoking in pregnancy is a well documented and potentially avoidable risk factor for a multitude of conditions, including miscarriage, low birth weight, perinatal death, childhood asthma and atopic disease [1-3]. Despite evidence-based knowledge of the harmful effects, tobacco smoking is still prevalent during pregnancy.

Prevalence studies in the 1980s showed that one in three pregnant women in Norway smoked during pregnancy, at that time among the highest smoking prevalence in Europe [4-6]. Eriksson et al. showed that the point prevalence of smoking at 18 weeks of gestation in Trondheim was 34% in 1987 compared to 22% in 1994, a statistically significant reduction. No effect of the national campaign against smoking during pregnancy launched in 1989 was found [7]. Public health interventions and smoking bans have since then shown success in some Western countries [8]. Norway has a history of more than 40 years of regulation of tobacco advertising and tobacco smoking in public. The 1975 Tobacco Act involved an advertising ban, 16 years age limit for buying tobacco products and labelling of tobacco products. Restrictions on smoking in public restaurants, bars, cafes, pubs and discotheques came in 1993, but a total ban on smoking in restaurants and bars first took effect on June 1st 2004. The first national comprehensive mass media campaign on tobacco and health for many years was accomplished during the study period in 2003.

A review article from 2000 stated that pregnancy and the postpartum period provide a window of opportunity to promote smoking cessation[9]. A Cochrane review from 2004 concluded that smoking cessation programmes in pregnancy reduce the proportion of women who continue to smoke [10]. Further, a meta analysis from 2000 found that a brief cessation counselling session of 5–15 minutes, when delivered by a trained provider with the provision of pregnancy specific self help materials, significantly increased rates of cessation among pregnant smokers, and these evidence based procedures were recommended to be adopted by all prenatal health care providers [11].

In 1997 the Norwegian Government appointed Trondheim as a model city to try out a new public intervention to counteract the rising incidence of asthma and allergic diseases. It was a prerequisite that the intervention programme should be possible to implement in ordinary pre- and postnatal care, without extra cost, and within normal consultation timeframe. The PACT-study was initiated in collaboration between the Norwegian University of Science and Technology (NTNU) and the municipality of Trondheim. The PACT study is a still ongoing, controlled, prospective, intervention study that was started in 2000 [12]. The primary objectives of the PACT study were to investigate the effectiveness of the risk-factor intervention on behavioural changes among parents, secondly to investigate the efficacy on the incidence of allergic diseases in the offspring from increasing omega-3- fatty acid intake and reducing parental smoking and indoor dampness [13].

The objectives of this study were to investigate parental smoking behaviour during pregnancy after introduction of a prenatal, structured, multi-disciplinary smoking cessation programme in primary care, and to compare smoking behaviour among pregnant women in the city of Trondheim with Bergen and Norway.

## Methods

The study was performed in the city of Trondheim, the capitol city in middle Norway with 160 000 inhabitants and approximately 2100 deliveries per year. The city holds a University with 20 000 students and 4500 employees. In all, 28 of 35 general practices (90 general practitioners), all eight community based midwifes and all 20 maternity health centres in Trondheim agreed to participate in the PACT study.

### Cohorts and subjects

**Figure 1:**
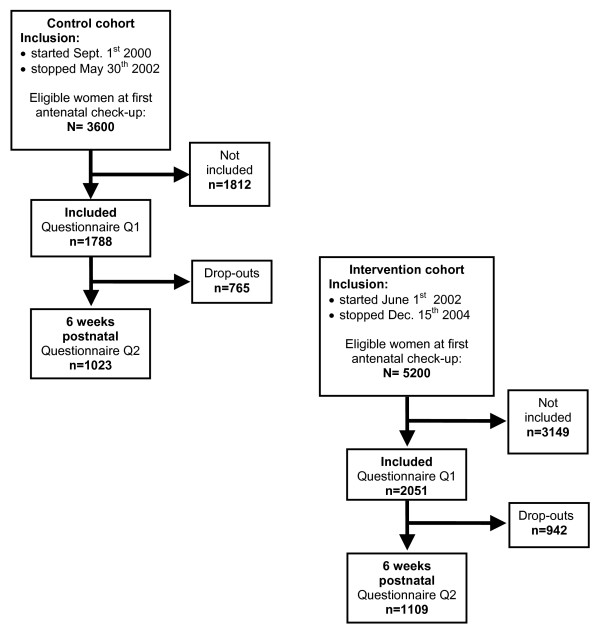
**Flow chart**. The same questionnaires were used in both cohorts. Q1 = questionnaire during pregnancy, measurement of smoking behaviour at start pregnancy and at inclusion at first antenatal check-up. Q2 = questionnaire at six weeks postnatal, measurement of present smoking behaviour.

Sequential birth cohorts were established to evaluate the intervention programme. From September 1^st ^2000 to May 30^th ^2002 all pregnant women who consulted their GPs or community based midwifes for pregnancy care were eligible to participate in the control cohort of the PACT study. Of some 3600 eligible pregnant women in Trondheim during this period, 1788 (50%) women were included and completed the pregnancy questionnaire (Q1), and 1023 (57%) of the participating women completed the questionnaire (Q2) six weeks after delivery (Figure 1). Participating women in the control cohort received common, nationwide recommended, advice on life-style, including smoking behaviour, following the routines each health-worker was familiar with at that time.

From June 1^st ^2002 to December 15^th ^2004 women were invited and included to the intervention cohort of the study. Of some 5200 pregnant women eligible to participate in the intervention cohort during this period, 2051 (40%) women gave their consent and answered the pregnancy questionnaire, and 1109 (54%) of the participating women completed the questionnaire six weeks after delivery.

All pregnant women were eligible to the PACT study if they were able to understand and fill in a questionnaire in Norwegian language with no other inclusion or exclusion criteria for either cohort.

### Intervention programme

The intervention programme on diet, indoor dampness, and smoking cessation was developed in collaboration with midwives, maternity care nurses, GPs, and parents as a multiple health behaviour intervention. The smoking intervention programme was a brief office intervention [14]. The intervention was adapted from the United States Department of Health and Human Services Public Health Service (USHPS) guideline "*Treating Tobacco Use and Dependence. Clinical Practice Guideline"*[15]. From June 2002 the intervention was adopted by the city health authorities to be implemented by all health professionals as an integrated part of the recommended maternity care life-style counselling programme in primary health care throughout Trondheim, regardless of participation in the PACT study or not. The intervention programme continued throughout pregnancy at GP and midwife consultations. The recommended primary care prenatal schedule for follow-up in Norway was the same for both cohorts and constitutes of 8–10 prenatal consultations with a GP or midwife from week 8–10 in pregnancy. This programme has been accessible to all women in Norway for many years, free of charge, and with an attendance rate of nearly 100%. The women were invited to bring their partners to the consultations, and if he was a smoker they were encouraged to make a smoking cessation effort together.

Midwifes, public health nurses and GPs were offered a three hours course to improve smoking cessation counselling skills, to obtain a consistent intervention and inspire enthusiasm [16]. All midwifes and 22 of the 28 participating group practices attended the course. In addition, all participating midwifes and GPs were supplied with written strategy guidelines describing the intervention in detail. Some 7% of the participating women in the intervention cohort were included by GPs that did not attend the three hours course. All women included in the intervention cohort were regarded as intervened upon whether their GP had delivered the intervention or not. Self-help materials to be offered to the participants were also distributed to all primary care health professionals. Continuous smoking cessation groups were allocated to the maternity care centres and administered by public health nurses. The health professionals received four follow-up newsletters during the intervention.

### Outcome variables

#### PACT data

The primary outcome variable was self-reported parental smoking behaviour at six weeks postnatal. The participants were asked to complete a self-reported life-style questionnaire including smoking behaviour at the first maternity clinic check-up (gestational week 8–12) and later at six weeks after delivery. Parental smoking during pregnancy was assessed with two questions at the antenatal questionnaire. The women were asked if they or their partner were smoking at the beginning of pregnancy, if they were smoking now and daily and/or weekly cigarette consumption. A separate question was asked about the total numbers of cigarettes smoked indoors. The same questions were asked six weeks postnatal. Smoking was coded as a dichotomous variable, if they were smoking more than one cigarette a week they were coded as smokers, if the answer was "no" they were coded as non-smokers, and if the answers to all questions on smoking were missing they were coded as missing. No biomarker such as hair nicotine was measured.

#### National data

Aggregated data from the Medical Birth Registry of Norway (MBR) were used to illustrate smoking cessation in Norway and the two comparable cities of Bergen and Trondheim from 1999 to 2004.

Bergen is the second largest city in Norway, with 245 000 inhabitants and around 3200 deliveries per year, with a University with some 16 000 students. Smoking data from the MBR were available from 1999–2004. These data are collected as a mandatory procedure at discharge from any maternity ward in Norway. Forms are completed by a midwife or physician interview and by using the hospital medical records. The women are asked if they smoked at the beginning or end of pregnancy, and they can answer "no", "occasionally" and "yes". Smoking was coded as a dichotomous variable, "occasionally" and "yes" were coded as smokers,"no" as non-smokers. Data were available for approximately 90% of the women who gave birth during the period from 1999 to 2004 according to information from the MBR.

#### The non-responder study

To investigate if there was a selection bias among participants in the PACT study we conducted an non-responder study where 391 parents who consecutively visited maternal postnatal care were asked to complete a short and anonymous questionnaire on age, socioeconomics, allergic disease and smoking behaviour, regardless of participation in the PACT-study or not.

#### Educational data

Maternal and paternal education was not accounted for in the original questionnaire. Thus, some 800 randomly selected parents answered questions on education (797 women and 812 men), either written or by telephone interview.

#### Approvals

The Regional Committee for Medical Research Ethics for Central Norway approved the study (Ref 120–2000). The study was granted license by the Norwegian Data Inspectorate to process personal health data and one of the parents signed a written informed consent formula (Ref 2003/953-3 KBE/-).

#### Statistics

SPSS for Windows^® ^ver.14.0 (Chicago, Ill. USA) was used for all statistical analyses. Comparisons between groups were tested by chi square tests for categorical data and independent t-tests for continuous data. Confidence intervals (95% CI) were estimated for prevalence and odds ratio using binomial distribution for dichotomous data, and normal distribution for continuous data. Confounding factors were identified by *a priori *knowledge, and maternal age at the beginning of pregnancy, parity; marital status, homeowner (as a proxy for social status) and paternal smoking at the beginning of pregnancy were tested in several models. The resulting set of covariates included maternal age at the beginning of pregnancy, parity and marital status. We used GLM with binomial regression in a predictive model (STATA ver. 10.0) to adjust smoking prevalence at the beginning of pregnancy, at inclusion and at 6 weeks post partum in both cohorts. Parental smoking was stratified into smokers and non-smokers at the beginning of pregnancy and at time of inclusion, and binary logistic regression models were used to estimate adjusted odds ratio (aOR) for smoking at inclusion and at six weeks postnatal, respectively, in the intervention cohort compared to the control cohort. Finally, binary logistic regression models were used to estimate aORs for the associations between smoking cessation before inclusion (spontaneous quitting) and background factors The results are analysed and presented according to the STROBE recommendations [17].

## Results

Some 28 of 35 general practices in Trondheim included a total of 2657 women into both cohorts by end 2004, ranging from 14 to 348 per practice, with 69% of the practices including more than 40 participants. The community midwives included altogether 1181 women. This gave a participation rate of about 44% of the eligible pregnant women in Trondheim. The non-responder study on 391 parents showed no selection bias for participants in the PACT-study regarding age, socioeconomics, allergic disease, or smoking behaviour (table 1).

**Table 1:** The non-responder study (N = 391). Characteristics of responders and non-responders to the PACT study

	Non-responders (n = 219)			Responders (n = 172)			
	n	%	CI 95%	n	%	CI 95%	p-value
Atopy in the family*	120	55.0	48.4–61.6	109	63.4	56.2–70.6	0.1
Mothers smoking at the beginning of pregnancy	46	21.0	15.6–26.4	28	16.3	10.8–21.8	0.25
Mothers smoking now	23	10.6	6.5–14.7	16	9.3	5.0–13.6	0.74
Fathers smoking at the beginning of pregnancy	39	18.6	13.5–23.8	32	18.9	13.1–24.8	1
Fathers smoking now	37	17.5	12.5–22.5	23	13.5	8.4–18.6	0.32
	Median	Mean	SD	Median	Mean	SD	p-value
Maternal age	30	30.8	5.1	30.5	30.7	4.8	0.89
Maternal education (years)	15	15.1	2.1	16	15.6	2.5	0.08
Fathers education (years)	15	15.1	3.1	16	15.3	2.9	0.64

*Atopy = mother or father or sibling reporting at least one atopic disease

### Background characteristics of the intervention and control cohorts

There were significantly more primiparous women, fewer single mothers, more educated women and more dropouts in the intervention cohort. The cohorts did not differ regarding maternal age, paternal education; the number of cigarettes smoked a day by mother or father, neither at the beginning of pregnancy nor at inclusion. The characteristics of the cohorts are presented in table 2.

**Table 2:** Characteristics of the intervention cohort (N = 2051) and the control cohort (N = 1788) at inclusion n = number of participants included in analysis

		Intervention cohort			Control cohort			
		n	%	95% CI	n	%	95% CI	p-value
Single mother*		1072	1.9	1.1–2.7	994	3.8	2.6–5.0	0.01
Primiparous		2051	56.6	54.5–58.7	1785	48.6	46.3–50.9	<0.001
		n	mean	SD	n	mean	SD	
Maternal age (years)		2044	28.6	4.6	1766	28.8	4.7	0.14
Maternal education (years)^†^		283	16.1	2.2	514	15.8	2.3	0.05
Paternal education^†^		289	15.4	2.7	523	15.2	2.9	0.34
No. of cig. a day among smokers at the beginning of pregnancy	Mother	462	8.6	7.9	475	8.0	6.1	0.19
	Father	438	9.8	8.3	413	9.6	6.7	0.68
No. of cig. a day among smokers at inclusion	Mother	140	5.3	7.4	184	4.9	4.0	0.57
	Father	355	8.8	8.6	356	8.2	6.4	0.27

*Data from questionnaire 6 weeks postnatal

^† ^Data available for 797 women and 812 men

Comparing dropouts from the intervention and control cohorts neither their mean age, 28.7 years (SD 4.8) and 28.5 years (SD 4.8) p = 0.56, nor being a homeowner, OR 1.1 (CI 95% 0.9–1.3) p = 0.43, nor the proportion who smoked more than 10 cigarettes a day, OR 1.1(CI 95% 0.7–1.6) did differ. There were, however, significantly more single women among dropouts in the intervention cohort, OR 1.3 (CI 95% 1.1–1.6) p = 0.004 (table 3). Among the women who smoked at inclusion, 140 and 184 women in the intervention cohort and control cohort, respectively, there was no significant difference in dropouts as 80 smokers dropped out from the intervention cohort and 90 smokers from the control cohort, (p = 0.15). Information on smoking among dropouts was missing for 7% and 5% in the intervention and control cohort, respectively.

**Table 3:** Maternal smoking prevalence among drop-outs

	Intervention cohort			Control cohort			
	n	%	95% CI	n	%	95% CI	p-value
Drop-outs*	942	45.9	42.7–49.8	765	42.8	39.3–46.3	0.05
Maternal smoking prevalence at the beginning of pregnancy	877	25.1	22.2–28.0	729	27.3	24.1–30.5	0.33
Maternal smoking prevalence during pregnancy	877	9.1	7.2–11.0	723	12.4	10.0–14.8	0.03

*Drop-outs = answered questionnaire in pregnancy but not answered questionnaire six weeks postnatal

### Smoking prevalence

The maternal smoking prevalence in the intervention cohort was significantly lower at the beginning of pregnancy and at inclusion, but not at six weeks post partum. Paternal smoking prevalence did not differ between the cohorts at the beginning of pregnancy, but was significantly lower in the intervention cohort at inclusion and six weeks post partum (table 4).

**Table 4:** Adjusted* parental smoking prevalence in the intervention cohort and the control cohort

	Intervention cohort		Control cohort				
Maternal smoking prevalence	%	95% CI	%	95% CI	aOR	95% CI	p-value
At the beginning of pregnancy	21.7	19.4–24.1	25.1	22.7–27.6	0.78	0.61–1.00	0.05
At inclusion	4.9	3.5–6.4	7.1	5.6–8.6	0.63	0.42–0.95	0.03
6 weeks postnatal	5.8	4.3–7.4	7.6	6.0–9.2	0.72	0.49–1.06	0.09
Paternal smoking prevalence							
At the beginning of pregnancy	21.9	19.2–24.6	24.7	21.8–27.5	0.86	0.69–1.07	0.17
At inclusion	17.0	14.5–19.5	21.2	18.5–23.9	0.76	0.60–0.97	0.03
6 weeks postnatal	14.5	12.2–16.9	17.9	15.4–20.4	0.78	0.60–1.00	0.05

*Parental smoking prevalence adjusted for maternal age at start pregnancy, first child and marital state.

### Smoking behaviour during pregnancy

Data stratified according to smoking behaviour at the beginning of pregnancy demonstrated that in the intervention cohort only one in four of the smoking women continued to smoke from the beginning of pregnancy until inclusion, with no significant difference between the cohorts. In contrast, most men continued to smoke in the same period, but significantly fewer in the intervention cohort. Very few men and women started smoking from the beginning of pregnancy until inclusion (table 5). In one model participants with missing smoking data were recoded as smokers. Neither in this model did we find any significant difference between the cohorts regarding smoking behaviour 6 weeks postnatal for women smoking at inclusion, aOR = 0.72 (95% CI 0.42–1.22, p = 0.22)

**Table 5:** Prevalence of parental smokers at inclusion stratified according to smoking behavior at the beginning of pregnancy

	Parental smoking prevalence at inclusion								
Smoking behavior at the beginning of pregnancy	Intervention cohort			Control cohort					
	n	%	95% CI	n	%	95% CI	aOR	95% CI	p-value
Mother non-smoking	1	0.1	0–0.8	1	0.1	0–0.9			
Mother smoking	57	24.7	19.5–30.6	82	31.7	26.3–37.6	0.66*	0.43–1.04	0.17
Father non-smoking	3	0.4	0.1–1.2	3	0.5	0–1.4			
Father smoking	162	75.3	69.2–80.7	176	84.6	79.1–88.9	0.58^†^	0.35–0.96	0.03

*Adjusted for first child, maternal age and paternal smoking at start pregnancy

^†^Adjusted for first child and maternal age

When we stratified according to smoking behaviour at inclusion we found that most women who smoked at inclusion continued smoking during pregnancy, about 7 in 10 women smoked at six weeks postnatal, with no significant difference between the cohorts. We found the same result among their partners. Some two percent of those who were non-smokers at inclusion were smoking at six weeks postnatal with no significant difference between the cohorts (table 6).

**Table 6:** Comparison of parental smoking between the cohorts after the smoking intervention programme

	Parental smoking prevalence 6 weeks post partum								
Smoking behaviour	Intervention cohort			Control cohort					
at inclusion:	n	%	95% CI	n	%	95% CI	aOR	95% CI	p-value
Mother non-smoking	20	2,1	1,3–3,2	27	3,2	2,2–4,6	0,77*	0,42–1,42	0,40
Mother smoking	42	72,4	59,1–83,3	57	67,9	57,3–76,9	1,54*	0,63–3,73	0,34
Father non-smoking	24	2,9	2,0–4,4	21	3,1	2,0–4,7	0,92^†^	0,53–1,59	0,76
Father smoking	116	69,9	62,5–76,4	134	74,0	67,2–79,9	0,61^†^	0,35–1,04	0,07

Stratified analysis according to smoking behaviour at inclusion, crude prevalence stated for smoking.

*aORs adjusted for maternal age, first child, and paternal smoking at the beginning of pregnancy

^†^aORs adjusted for first child and maternal age

When we looked at both cohorts combined women who were at risk for continued smoking after the beginning of pregnancy and still smoking at inclusion were living single, multiparous women and women who smoked more than 10 cigarettes a day. At the beginning of pregnancy 518 (25%) of the women were smoking more than one cigarette weekly, 25 women had missing data, and 493 women were included in the analysis (table 7). At high risk for continued smoking at inclusion were multiparous women smoking > = 10 cigarettes a day compared to all other smoking women at the beginning of pregnancy, OR 3.5, p < 0.001.

**Table 7:** Logistic regression of background factors predicting maternal smoking at inclusion* among pre-pregnancy smokers

	Adjusted odds ratio for smoking at inclusion		
	aOR	95% CI	p-value
Maternal age < = 24 years vs.	reference		
> 31 years	1.57	0.82–3.02	0.18
Primiparous vs.	reference		
multiparous	1.71	1.09–2.69	0.02
Married or cohabitant vs.	reference		
living single	3.01	1.43–6.34	0.004
Mother smoking <= 10 cig. a day vs.	reference		
> 10 cig. a day	3.07	2.04–4.64	< 0.001

*(1 = smoking, n = 150) (0 = non-smoking = spontaneous quitters, n = 362)

### Indoor smoking

At inclusion 18% of the parental smokers in the intervention cohort, and 28% in the control cohort reported indoor smoking (p = 0.01). At six weeks post partum only one parent in the intervention cohort and nine parents (5%) of the parental smokers reported indoor smoking (p = 0.04). When all with missing data on indoor smoking were recoded as indoor smokers 5% and 8% were indoor smokers in the intervention cohort, and control cohort, respectively.

### Smoking cessation in Trondheim, Bergen and Norway

**Figure 2:**
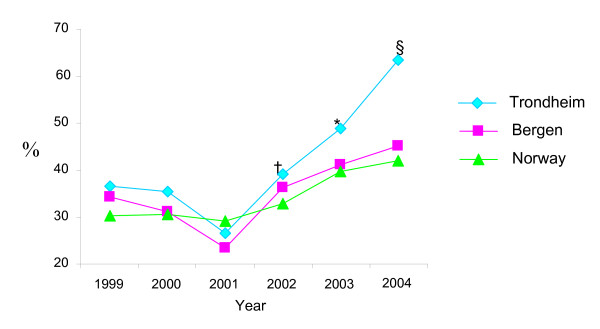
**Proportion of women who stopped smoking during pregnancy in Trondheim, Bergen and Norway 1999–2004**. Data from the Medical Birth Registry in Norway. ^†^p = 0.43 for difference in smoking cessation between Trondheim and Bergen 2002. *p = 0.03 for difference in smoking cessation between Trondheim and Bergen 2003. ^§^p = < 0.001 for difference in smoking cessation between Trondheim and Bergen 2004.

Data from MBR showed a quitting rate of about 30–40% from 1999 to 2002 with no difference between Trondheim, Bergen and Norway. In 2003 and 2004 the proportion of women who stopped smoking during pregnancy in Trondheim increased seemingly more than in Bergen and Norway (figure 2). In Trondheim 64% of the pre-pregnancy smoking women stopped smoking at the beginning of pregnancy or during pregnancy in 2004.

## Discussion

We found a low smoking prevalence at inclusion, 4.9% and 7.1% in the intervention cohort and the control cohort, respectively. Only a quarter of the pre-pregnancy smoking women still smoked at inclusion time with no difference between the cohorts. During the intervention period from inclusion until six weeks postnatal, 7 in 10 smokers still smoked six weeks postnatal with no significant difference between the cohorts. At inclusion 18% and 28% reported indoor smoking in the intervention cohort and the control cohort, respectively. At six weeks postnatal very few of the smokers reported indoor smoking, only one parent in the intervention cohort, and nine parents in the control cohort.

Data from MBR illustrating quitting rates in Trondheim, Bergen and Norway showed a seemingly higher proportion of women who stopped smoking at the beginning or during pregnancy in Trondheim than in Bergen in 2003 and 2004.

The study had a controlled design comparing sequential total and unselected cohorts of pregnant women from the beginning of pregnancy until six weeks postnatal. Choosing a controlled design including whole birth cohorts made it possible in a real life approach to test the intervention programme. The assessment of smoking behaviour was consistent through the observation period and across cohorts, and independent of clinical practice. Furthermore, the majority of care providers were trained and motivated to deliver the recommended intervention modalities on repeated occasions both to those who smoked and those who had quit smoking [18]. Finally, when health professionals take part in a scheduled and structured intervention, it may counteract any potential negative beliefs and attitudes against promoting smoking cessation [19]. The possibility to compare smoking cessation nationally and in the two comparable university cities of Trondheim and Bergen in the same period that the sequential cohorts in PACT were investigated was an additional strength of the study.

The one year time difference between the control cohort and intervention cohort might have biased the results towards a better effect of the intervention due to secular trends. However, this was the design of choice primarily because a public and community based intervention including the entire primary health care in the municipality would be impossible to implement without contaminating a co-existing control cohort. Secondly, comparing total birth cohorts also ensured high conformity between the cohorts regarding population size, race/ethnicity, maternal educational level, income, environment, urbanization and social characteristics [20]. The use of self reported questionnaires on smoking behaviour were adopted based on documentation indicating equal or better reliability compared to interviews using a structured questionnaire [21,22]. Furthermore, a Norwegian validation study had already shown that Norwegian pregnant women generally reported their smoking habits correctly [23]. We used no biomarkers for tobacco smoking, as this is unfeasible in large epidemiologic studies, and earlier studies have demonstrated that such biomarkers give little or no additional accuracy to the registration of smoking behaviour when compared to self reported smoking in pregnancy [24,25].

### Participation and dropouts

During the study period starting September 1^st ^2000 and ending December 15^th ^2004, 3839 of some 8800 eligible pregnant women in Trondheim took part in the PACT study, giving a participation rate of 44%. The participation rate was a consequence of low inclusion activity among many GPs and midwifes, and not a consequence of self selection among women. There is no reason to assume a selection bias, as confirmed by results from the non-responder study which included 391 subjects.

Of the 3839 women that were included during pregnancy, 2132 (56%) answered the questionnaire six week postnatal. This is a high loss to follow-up, and most probably due to forgetfulness or failing routines for follow-up among the health professionals. One would also expect a certain degree of exhaustion among GPs and midwives in a study of such longevity [26]. If the loss to follow-up is assigned to forgetfulness or low attention during follow-up both among participants and health professionals, it may be assumed that the participants are lost at random. This is supported by the observation that baseline characteristics between dropouts in the two cohorts only differed for single mothers. If so, even a loss to follow-up of 60% is shown not to represent important bias [27]. Importantly, we had almost no active withdrawals in either cohort.

We found no significantly reduced parental smoking prevalence in the intervention cohort six weeks postnatal when we performed a stratified analysis according to smoking behaviour at inclusion. The high quitting rate observed in both cohorts was apparently due to spontaneous quitting before inclusion. Therefore only a hardcore of resilient smokers were left to intervene on, women who had taken their choice of continued smoking during pregnancy probably despite knowledge of the harmful effects and social stigma. In this respect multiparous women who smoked more than 10 cigarettes a day were at highest risk. This is in agreement with results from several other smoking intervention studies in pregnancy [28,29].

We found a very low prevalence of reported indoor smoking in both cohorts which may indicate that there was awareness in both cohorts of the harmful effect of SHS on small children, but answering according to social desirability may also explain this result.

### Smoking cessation in Trondheim and Bergen

The MBR aggregated data showed a higher quitting rate during pregnancy in Trondheim than in Bergen after the intervention programme in the PACT study commenced. The MBR data for Trondheim comprise both women participating in the PACT study and non-participating women. The women in the two cities had been exposed for the same national legislation and anti smoking campaigns. What differ between the two cities are the PACT study and the fact that the intervention programme was adopted as an integrated part of the recommended maternity care life-style counselling programme throughout Trondheim. An interpretation may be that the PACT study in this way have increased the attention on the health hazards of smoking in pregnancy, both among GPs and midwifes, but also among the parents to be, and in this way brought about the significantly higher smoking cessation rate observed in the MBR data for Trondheim compared to Bergen.

## Conclusion

A new smoking intervention programme as part of a multiple health behaviour intervention did not reduce parental smoking prevalence during pregnancy in the intervention cohort compared to the control cohort. Most women were spontaneous quitters and gave up smoking early in pregnancy before the intervention took place. We found a low indoor smoking prevalence in both cohorts, which may reflect a high degree of awareness of the harmful effects of smoking during pregnancy. Data from the MBR showed a higher quitting rate in Trondheim compared to Bergen in 2003 and 2004 which may have been facilitated by the supplemental attention on smoking behaviour the PACT study initiated.

## Competing interests

The authors declare that they have no competing interests.

## Authors' contributions

TØ and OS participated in the design and coordination of the study and drafted the manuscript. JAJ participated in the design of the study and performed statistical analysis. RJ conceived of the study, and participated in its design and coordination and helped draft the manuscript. All authors read and approved the final manuscript.

## Pre-publication history

The pre-publication history for this paper can be accessed here:



## References

[B1] Alati R, Al MA, O'Callaghan M, Najman JM, Williams GM (2006). In utero and postnatal maternal smoking and asthma in adolescence. Epidemiology.

[B2] Kulig M, Luck W, Lau S, Niggemann B, Bergmann R, Klettke U (1999). Effect of pre- and postnatal tobacco smoke exposure on specific sensitization to food and inhalant allergens during the first 3 years of life. Multicenter Allergy Study Group, Germany. Allergy.

[B3] Lannero E, Wickman M, Pershagen G, Nordvall L (2006). Maternal smoking during pregnancy increases the risk of recurrent wheezing during the first years of life (BAMSE). Respir Res.

[B4] Cnattingius S, Haglund B (1997). Decreasing smoking prevalence during pregnancy in Sweden: the effect on small-for-gestational-age births. Am J Public Health.

[B5] Dodds L (1995). Prevalence of smoking among pregnant women in Nova Scotia from 1988 to 1992. CMAJ.

[B6] Haug K, Aaro LE, Fugelli P (1992). Smoking habits in early pregnancy and attitudes towards smoking cessation among pregnant women and their partners. Fam Pract.

[B7] Eriksson KM, Salvesen KA, Haug K, Eik-Nes SH (1996). Smoking habits among pregnant women in a Norwegian county 1987–1994. Acta Obstet Gynecol Scand.

[B8] Spinney L (2007). Public smoking bans show signs of success in Europe. Lancet.

[B9] Diclemente CC, Dolan-Mullen P, Windsor RA (2000). The process of pregnancy smoking cessation: implications for interventions. Tob Control.

[B10] Lumley J, Oliver SS, Chamberlain C, Oakley L (2004). Interventions for promoting smoking cessation during pregnancy. Cochrane Database Syst Rev.

[B11] Melvin CL, Dolan-Mullen P, Windsor RA, Whiteside HP, Goldenberg RL (2000). Recommended cessation counselling for pregnant women who smoke: a review of the evidence. Tob Control.

[B12] Jenssen J, Storrø O, Øien T, Johnsen R (2001). Prevention of allergy among children in Trondheim. Allergi i praksis.

[B13] Peat JK (1996). Prevention of asthma. Eur Respir J.

[B14] Hughes JR (2003). Motivating and helping smokers to stop smoking. J Gen Intern Med.

[B15] Fiore MC, Bailey WC, Cohen S (2000). Treating Tobacco Use and Dependence. Clinical Practice Guideline. US Department of Health and Human Services Public Health Service: June 2000.

[B16] Thompson SC, Schwankovsky L, Pitts J (1993). Counselling patients to make lifestyle changes: the role of physician self-efficacy, training and beliefs about causes. Fam Pract.

[B17] von EE, Altman DG, Egger M, Pocock SJ, Gotzsche PC, Vandenbroucke JP (2007). The Strengthening the Reporting of Observational Studies in Epidemiology (STROBE) statement: guidelines for reporting observational studies. Prev Med.

[B18] Garg A, Serwint JR, Higman S, Kanof A, Schell D, Colon I (2007). Self-efficacy for smoking cessation counseling parents in primary care: an office-based intervention for pediatricians and family physicians. Clin Pediatr (Phila).

[B19] Vogt F, Hall S, Marteau TM (2005). General practitioners' and family physicians' negative beliefs and attitudes towards discussing smoking cessation with patients: a systematic review. Addiction.

[B20] Kaneko M (1999). A methodological inquiry into the evaluation of smoking cessation programmes. Health Educ Res.

[B21] Okamoto K, Ohsuka K, Shiraishi T, Hukazawa E, Wakasugi S, Furuta K (2002). Comparability of epidemiological information between self- and interviewer-administered questionnaires. J Clin Epidemiol.

[B22] Shibata A, Matsuo M, Fukuda K (2002). Validity of the responses to self-administered questionnaires as compared with the responses to interviews using a structured questionnaire. Kurume Med J.

[B23] Nafstad P, Kongerud J, Botten G, Urdal P, Silsand T, Pedersen BS (1996). Fetal exposure to tobacco smoke products: a comparison between self-reported maternal smoking and concentrations of cotinine and thiocyanate in cord serum. Acta Obstet Gynecol Scand.

[B24] McDonald SD, Perkins SL, Walker MC (2005). Correlation between self-reported smoking status and serum cotinine during pregnancy. Addict Behav.

[B25] Pickett KE, Rathouz PJ, Kasza K, Wakschlag LS, Wright R (2005). Self-reported smoking, cotinine levels, and patterns of smoking in pregnancy. Paediatr Perinat Epidemiol.

[B26] Galea S, Tracy M (2007). Participation rates in epidemiologic studies. Ann Epidemiol.

[B27] Kristman V, Manno M, Cote P (2004). Loss to follow-up in cohort studies: how much is too much?. Eur J Epidemiol.

[B28] Secker-Walker RH, Solomon LJ, Flynn BS, Skelly JM, Lepage SS, Goodwin GD (1995). Smoking relapse prevention counseling during prenatal and early postnatal care. Am J Prev Med.

[B29] Solomon L, Quinn V (2004). Spontaneous quitting: self-initiated smoking cessation in early pregnancy. Nicotine Tob Res.

